# Caveat Medicus: It’s Time to Re-Think Stratification, You May Not Be Helping

**DOI:** 10.1177/11772719231174746

**Published:** 2023-05-14

**Authors:** Ognjen Arandjelović

**Affiliations:** University of St Andrews, St Andrews, UK

**Keywords:** Risk, precision, personalized, targeted, biomarkers

## Abstract

**Background:**

The focus of the present Letter is on the large and seemingly fertile body of work captured under the umbrella of ‘patient stratification’.

**Objectives:**

I identify and explain a fundamental methodological flaw underlying the manner in which the development of an increasingly large number of new stratification strategies is approached.

**Design:**

I show an inherent conflict between the assumptions made, and the very purpose of stratification and its application in practice.

**Methods:**

I analyse the methodological underpinnings of stratification as presently done and draw parallels with conceptually similarly flawed precedents which are now widely recognized.

**Results:**

The highlighted flaw is shown to undermine the overarching ultimate goal of improved patient outcomes by undue fixation on an ill-founded proxy.

**Conclusion:**

I issue a call for a re-think of the problem and the processes leading to the adoption of new stratification strategies in the clinic.

## Introduction

Much work in the realm of clinical pathology is concerned with the problem of *patient stratification*,^1-4^ to wit, the process of categorizing patients on an individual level according to some measure of risk, for example, risk of death. The premise behind this effort is sound enough at the first sight and is simple to understand: by identifying which patients are at higher and which at lower risk, the always scarce resources can be utilized best and targeted towards those in most urgent need, thus delivering improved overall patient outcomes in the context the real world and the limitations imposed by practical considerata.^5^ Moreover, effective risk stratification can prevent over-treatment and unnecessary side effects, thus improving overall quality of life of affected patients.

**Figure 1.**
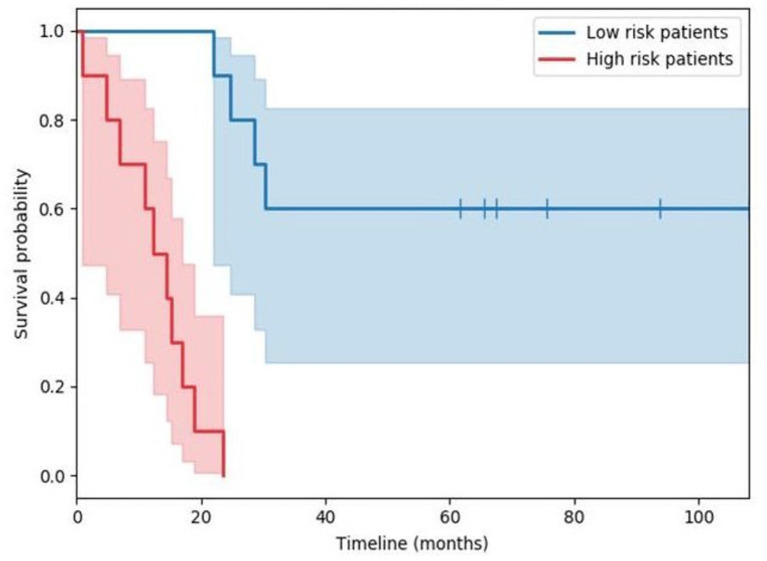
Example of the Kaplan-Meier derived estimates of 2 survival functions (red and blue lines) evidencing patient stratification. The corresponding shared areas indicate the standard deviations of the estimates across time.

The emergence of digital pathology, that is the high- resolution digitization of glass-mounted histopathological specimens, is becoming more commonplace in the clinic, and has led to a wide range of new opportunities for stratification improvement. New technologies, such as brightfield^6^ or multiplexed immunofluorescence^7^ facilitate the identification, classification and quantification of multiple cell types or biomarkers co-localized at the single-cell resolution and on a single tissue section; the concurrent advances in computation, most significantly in artificial intelligence and machine learning open yet further doors to more sophisticated and personalized risk assessment and thus stratification by means of either better utilization of existing prognostic variables or the discovery of novel, previously unknown ones.^8^ Yet, the fundamental premises of the very idea of patient stratification remain unchallenged, with the ultimate aim thereof, to wit, the improvement of patient outcomes, slipping away from the primary focus and being replaced with the proxy goal of greater separation of patient strata; see Figure 1. With the present Letter, my goal is to remedy this and highlight an inherent flaw in the fixation on stratification in its own right, thus raising awareness of the potential damage which may be done to suffering individuals, and the loss of research effort and time, and call for a re-evaluation of the entire approach.

## The Methodological Flaw

The key observation that leads to the appreciation of the methodological fallacy at the crux of the process is that many new stratification approaches are *developed post hoc*, that is, without the said stratification informing differential treatment across the cohort; this is obvious, in that the stratification has to be based on the measurement of the outcomes first and hence having to follow it. Yet, this stratification is expected to be *employed* precisely so as to tailor treatment in a more personalized fashion, thus changing the outcomes in practice.

Let me concretize this so that the importance of what is said is properly understood. Consider a patient who is identified by a stratification method as belonging to a high risk group due to their short life expectancy effected by a disease. Prioritizing the treatment of this patient may not be a sensible option because it may well be that this patient’s poor prognosis is the result of a state which is not improvable by the available treatment options; in other words, they will die soon treatment or no treatment, prioritized or not. Put differently, there may be a correlation between patients’ strata assignments and their treatability, that is the potential for improving the ultimate outcomes of interest. This observation conflicts the at present largely unrecognized implicit assumption underlying the current approach to patient stratification research, which is that patient survivability in the absence of treatment, or given patient-undifferentiated treatment, is independent of their treatability, to say nothing of their treatability by specific means. Ultimately, the problem of stratification cannot be divorced of the particularities of a specific treatment, that is, stratification must be tied and conditioned on the specific treatments that would be provided to the different strata. Without this being done, any stratification is inherently insufficiently informed and its proxy benefits must be further re-assessed by examining the ultimate outcome of interest, which is to say the survival of stratified patients *after* stratification informed treatment is provided to them.

An insightful conceptual parallel can be drawn here with the vocal emphasis on early screening for various cancers.^9^ We observe a similar pattern to that which was described *ut supra*: there is abundant evidence that early screening significantly increases survival rates at key monitoring intervals; hence, it is seen as a means of improving patient longevity. Yet, the inference is underlain by a serious statistical flaw or a similar nature as that which I elucidated earlier. Not only does early screening by the very fact that it happens earlier, increase survival rates *irrespective* of any changes to the patients’ condition (which, to be fair, is sometimes accounted for by the authors of studies), it is also the case that earlier screening detects changes which *appear* like cancer under the microscope but in fact never end up developing into a symptomatic disease, let alone one which shortens one’s life.^10^

## Conclusions

In this Letter, I identified and drew the attention to a major methodological flaw in the large body of work in clinical pathology falling under the umbrella of patient stratification. I explained how the assumptions used to derive stratification models *inherently* conflict with the very purpose of such models and the manner in which they are employed in practice, leaving the resultant sequacious focus on the development of ‘better’ stratification strategies ill-founded and ultimately unconducive to the optimal improvement in patient outcomes. The elucidation of the fundamental methodological error at the heart of the issue, should serve a means of guiding the direction of change needed to rectify the problem. One possibility is to ensure that any stratification undergoes a multi-stage process not unlike that required for the approval of new drugs, for example, with the first stage being similar to what is currently found in the literature, and a follow-up stage which assesses performance in terms of the ultimate goal of interest: patient outcomes in actual clinical practice which involves any treatment choices consequent on the stratification assessed.

## Declarations

**Ethics Approval and Consent to Participate:** Not applicable.

**Consent for Publication:** Not applicable.

**Availability of Data and Materials:** Not applicable.
